# Stabilization of Nucleosomes by Histone Tails and by FACT Revealed by spFRET Microscopy

**DOI:** 10.3390/cancers9010003

**Published:** 2017-01-06

**Authors:** Maria E. Valieva, Nadezhda S. Gerasimova, Kseniya S. Kudryashova, Anastasia L. Kozlova, Mikhail P. Kirpichnikov, Qi Hu, Maria Victoria Botuyan, Georges Mer, Alexey V. Feofanov, Vasily M. Studitsky

**Affiliations:** 1 Biology Faculty, Lomonosov Moscow State University, Leninskie Gory 1, Moscow 119992, Russia; durnopeyko.maria@gmail.com (M.E.V.); gerasimova@mail.bio.msu.ru (N.S.G.); rekamoskva@mail.ru (K.S.K.); mika.lorens@yandex.ru (A.L.K.); kirpichnikov@inbox.ru (M.P.K.); 2 Shemyakin-Ovchinnikov Institute of Bioorganic Chemistry of Russian Academy of Sciences, Moscow 117997, Russia; 3 Department of Biochemistry and Molecular Biology, Mayo Clinic, Rochester, MN 55905, USA; Hu.Qi@mayo.edu (Q.H.); botuyan.maria@mayo.edu (M.V.B.); Mer.Georges@mayo.edu (G.M.); 4 Fox Chase Cancer Center, Philadelphia, PA 19111, USA

**Keywords:** FACT, facilitates chromatin transcription, Spt16, SSRP1, nucleosome

## Abstract

A correct chromatin structure is important for cell viability and is tightly regulated by numerous factors. Human protein complex FACT (facilitates chromatin transcription) is an essential factor involved in chromatin transcription and cancer development. Here FACT-dependent changes in the structure of single nucleosomes were studied with single-particle Förster resonance energy transfer (spFRET) microscopy using nucleosomes labeled with a donor-acceptor pair of fluorophores, which were attached to the adjacent gyres of DNA near the contact between H2A-H2B dimers. Human FACT and its version without the C-terminal domain (CTD) and the high mobility group (HMG) domain of the structure-specific recognition protein 1 (SSRP1) subunit did not change the structure of the nucleosomes, while FACT without the acidic C-terminal domains of the suppressor of Ty 16 (Spt16) and the SSRP1 subunits caused nucleosome aggregation. Proteolytic removal of histone tails significantly disturbed the nucleosome structure, inducing partial unwrapping of nucleosomal DNA. Human FACT reduced DNA unwrapping and stabilized the structure of tailless nucleosomes. CTD and/or HMG domains of SSRP1 are required for this FACT activity. In contrast, previously it has been shown that yeast FACT unfolds (reorganizes) nucleosomes using the CTD domain of SSRP1-like Pol I-binding protein 3 subunit (Pob3). Thus, yeast and human FACT complexes likely utilize the same domains for nucleosome reorganization and stabilization, respectively, and these processes are mechanistically similar.

## 1. Introduction

In eukaryotes, epigenetic information is encoded in chromatin and used for genome regulation and stability [1]. A correct chromatin structure is important for cell viability and is highly regulated by numerous factors. Histone chaperones play a key role in this process. FACT (facilitates chromatin transcription) is a protein complex with a wide range of functions: it is a histone chaperone that plays an important role in cell differentiation, transcription, DNA replication, DNA repair, and tumorigenesis [1]. FACT is overexpressed in some types of cancer cells in vitro [2] and in vivo [3]. It is a target for anticancer drugs called curaxins [2,4]. As a histone chaperone, FACT helps to preserve chromatin structure which, in turn, prevents transcription initiation from cryptic promoters [5,6,7]. FACT also plays a role in nucleosome survival during transcript elongation by RNA polymerase II, stabilizing the proximal or distal H2A-H2B dimer(s) [8].

**Figure 1:**
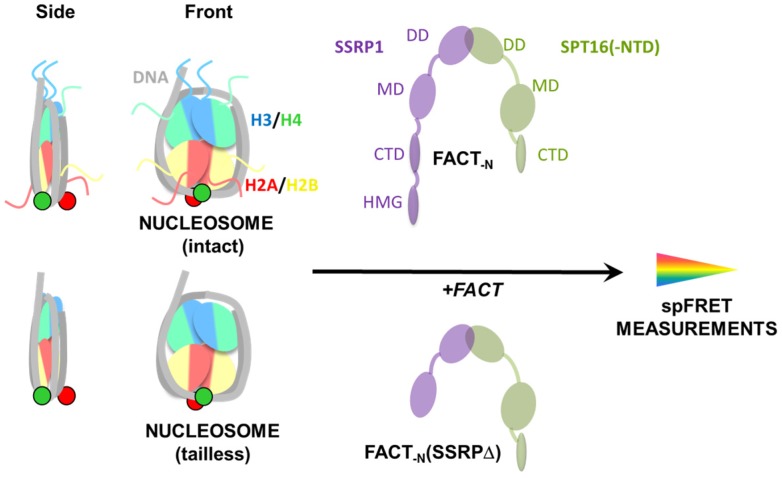
Experimental approach for analysis of the effect of human FACT on nucleosome structure and dynamics.

In animal and plants, FACT is a heterodimer formed by two subunits: a suppressor of Ty 16 (Spt16) and a structure-specific recognition protein 1 (SSRP1). SSRP1 and Spt16 are multi-domain proteins, and both of them contain dimerization domains (DD), middle domains (MD), and acidic C-terminal domains (CTD). Spt16 has an additional N-terminal domain (NTD), while SSRP1 has a high mobility group (HMG) domain on the C-terminus (Figure 1) [9]. Spt16-NTD interacts with H3-H4 tetramers [10]. The Spt16-MD of *Chaetomium thermophilum* binds H2A-H2B dimers with nanomolar affinity [11], and interacts with H3-H4 tetramers with micromolar affinity through a different interface [11,12]. The Spt16:H2A-H2B complex is similar in structure to the human ANP32E:H2A.Z-H2B complex [13,14,15]. ANP32E (acidic nuclear phosphoprotein 32 kDa) binds a histone variant, H2A.Z, through its acidic domain and this interaction is important for H2A.Z deposition on promoter regions. Similarly, CTD of Spt16 is involved in H2A-H2B binding by FACT [16] and participates in nucleosome reorganization [17].

In the present study, fluorescently labeled mononucleosomes were studied using single-particle Förster resonance energy transfer (spFRET) microscopy to characterize the nucleosome-stabilizing activity of FACT. Previously, this approach was found to be very informative for yeast FACT (yFACT) [18]. While yFACT was shown to uncoil nucleosomes, the recombinant human FACT has a nucleosome-stabilizing activity. Here we describe and compare the interactions of intact nucleosomes and nucleosomes destabilized by trypsin cleavage of histone tails with recombinant human FACT and its mutant with a truncated SSRP1 subunit.

## 2. Results

### 2.1. Experimental System

**Figure 2:**
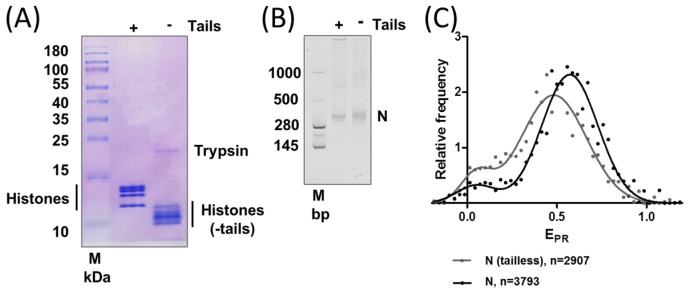
Removal of histone tails changes the structure of nucleosomes. (**A**) Removal of histone tails by trypsin changes their mobilities in the denaturing gel; (**B**) Intact and tailless nucleosomes have similar mobilities in a native gel; (**C**) Typical frequency distributions of proximity ratios (E_PR_) for intact (N) and tailless nucleosomes (N(tailless)). Analysis by single-particle Förster resonance energy transfer (spFRET) microscopy. *n* is the number of analyzed nucleosomes.

To evaluate the effect of human FACT on the structure of nucleosomal DNA, mononucleosomes containing DNA labeled with a single donor-acceptor pair of fluorophores were utilized (Figure 1). Nucleosomes were assembled using either intact or tailless chicken histones (Figure 1) and DNA containing a previously characterized 603 nucleosome positioning sequence and 20 bp linker DNA [18,19,20]. The fluorophores fluorescein (FAM) and carboxy-X-rhodamine (ROX) were introduced at the previously characterized positions +35 and +112 bp relative to the 603 nucleosome positioning sequence boundary [21]. In intact nucleosomes, fluorophores were positioned in adjacent gyres near the contact between H2A-H2B dimers (Figure 2A).

Activities of three protein complexes were compared in the present study: a complex without the NTD of Spt16 (hereinafter referred to as FACT_-N_), FACT_-N_ without the CTD and HMG domains of SSRP1 (FACT_-N_(SSRP∆)) and FACT_-N_ without both acidic domains (CTD of Spt16 and CTD-HMG of SSRP1, FACT_-N_(Spt6∆/SSRP∆)). The NTD-less complex is the largest full-size human FACT that can be currently obtained using recombinant protein technology. The absence of the NTD does not cause lethality in yeast cells, possibly because the functions of NTD of Spt16 and MD of Pol I-binding protein 3 (Pob3), an SSRP1-like subunit of yFACT, partially overlap [16].

Fluorescently labeled mononucleosomes containing either intact or tailless core histones (positions of FAM and ROX labels are shown by red and green circles, respectively) were incubated with FACT_-N_ (recombinant NTD-less variant) or its mutant (FACT_-N_(SSRP∆)) and studied by spFRET microscopy to reveal the effect of FACT on the structure of nucleosomal DNA. FRET efficiency decreases when the distance between labeled DNA sites increases (and vice versa), allowing the analysis of FACT’s influence on the proximity of the labeled gyres of nucleosomal DNA in single nucleosomes.

A laser with a 488 nm wavelength was used to excite the donor fluorophore (FAM) of single nucleosomal complexes in solution, when they diffused freely across the focal volume of a microscope. Fluorescence intensities of both donor (FAM) and acceptor (ROX) fluorophores were measured as described previously [22]. Using these intensities, the proximity ratios (E_PR_) were calculated and compared in the absence or presence of FACT_-N_ or FACT_-N_(SSRP∆) to reveal the changes in FRET efficiency and, therefore, in the distance between labeled DNA sites [18].

### 2.2. Removal of Histone Tails Affects the Structure of Nucleosomes

To obtain tailless nucleosomes, the chromatin from chicken erythrocytes was cleaved by trypsin. The analysis of trypsin-cleaved histones (Figure 2A) revealed that all core histones were cleaved to the expected extent that was achieved and extensively characterized previously for histones from chicken erythrocytes [23] and human histones [24].

Fluorescently labeled nucleosomes reconstituted with tailless or intact core histones were analyzed by native polyacrylamide gel electrophoresis (PAGE) (Figure 2B). The mobilities of both types of nucleosomes in a native gel were similar, but the band corresponding to tailless nucleosomes was more diffuse, probably indicating the existence of a broader range of conformations of tailless nucleosomes in the native gel.

Structures of intact and tailless nucleosomes were compared by spFRET microscopy. Both types of nucleosomes are characterized by E_PR_ curves that have two peaks each (Figure 2C) and can be described by the superposition of two Gaussians. A minor peak with low E_PR_ likely corresponds to a fraction of free DNA or unfolded nucleosomes with a large distance between the labels. The latter explanation is more likely, since no free DNA is observed in the native gel (Figure 2B). A major peak with high E_PR_ corresponds to compact nucleosomes (~85% of all nucleosomes) with a close proximity of labels on DNA. This result is in good agreement with data published previously for nucleosomes labeled with Cy3 and Cy5 fluorophores in the same +35 and +112 positions on nucleosomal DNA [18]. As compared to intact nucleosomes, the tailless ones have a smaller fraction of compact nucleosomes, and their high-E_PR_ peak is shifted to lower values (Figure 2C). This shift indicates that DNA folding on the octamer of tailless histones is disturbed in comparison to intact nucleosomes.

**Figure 3:**
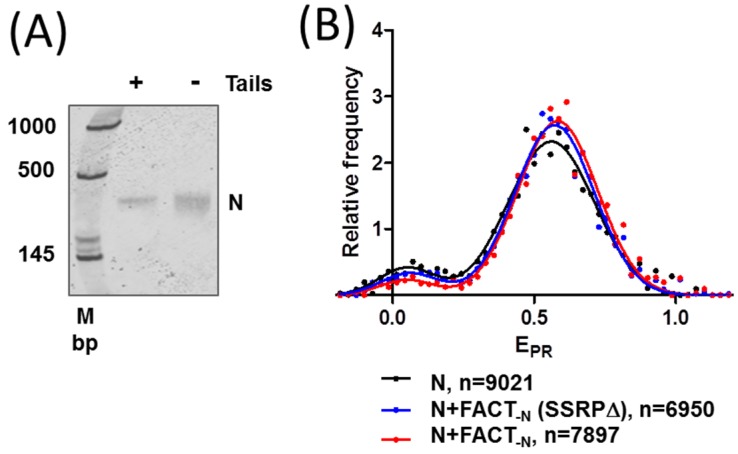
FACT minimally affects the structure of intact nucleosomes. (**A**) Gel-purified intact and tailless nucleosomes have similar mobilities in a native gel; (**B**) Intact nucleosomes are minimally affected by a complex without the N-terminal domain (NTD) of Spt16 (FACT_-N_) or by FACT_-N_ without the C-terminal domain (CTD) and high mobility group (HMG) domain of structure-specific recognition protein 1 (SSRP1) (FACT_-N_(SSRPΔ)). Analysis by spFRET microscopy. *n* is the number of analyzed nucleosomes.

In order to study the effect of FACT on the structure of the nucleosomes, 603 nucleosomes were gel-purified from an excess of donor chromatin used for nucleosome reconstitution. The integrity and purity of the nucleosomes was confirmed by native PAGE (compare gels on Figure 2B and Figure 3A). spFRET analysis revealed that the purification procedure did not affect the difference in the structures of intact and tailless nucleosomes (Supplementary Materials Figure S1).

### 2.3. FACT Minimally Affects the Structure of Intact Nucleosomes

The addition of FACT_-N_ or FACT_-N_(SSRPΔ) to intact nucleosomes induced minimal changes in the frequency distributions of E_PR_ (Figure 3B, Table 1). According to the unpaired two-tailed *t* test, the changes in low- and high-E_PR_ fractions as well as in positions of E_PR_ maxima of nucleosomes in the presence of FACT_-N_ or FACT_-N_(SSRPΔ) were not significant. Thus, neither FACT_-N_ nor FACT_-N_(SSRPΔ) affected the structure of intact nucleosomes.

The FACT mutant missing the CTD and HMG domains of SSRP1 and CTD of Spt16 induced fast and extensive aggregation of nucleosomes detected as extremely strong signals by spFRET (not shown).

### 2.4. FACT Stabilizes Tailless Nucleosomes

**Figure 4:**
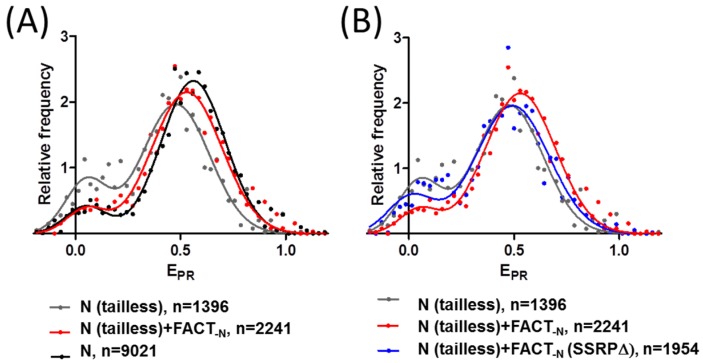
FACT stabilizes tailless nucleosomes. CTD and/or HMG domain(s) of SSRP1 are required for this activity. Effect of FACT_-N_ (**A**) and comparison of the effects of FACT_-N_ and FACT_-N_(SSRPΔ) on tailless nucleosomes (**B**) as detected by spFRET microscopy. Typical frequency distributions of proximity ratios (E_PR_) are shown. *n* is a number of analyzed nucleosomes.

The addition of FACT_-N_ to tailless nucleosomes resulted in significant (*p* < 0.05) changes in the frequency distribution of E_PR_: a decrease in the low-E_PR_ fraction and a shift of the high-E_PR_ maximum from 0.49 to 0.54 (Figure 4A, Table 1). Both types of changes can be interpreted as FACT-induced stabilization of a nucleosome structure. The E_PR_ distributions of tailless and intact nucleosomes in the presence of FACT_-N_ are similar except for a small difference in the position of the high-E_PR_ maxima (Figure 4A). It seems that FACT_-N_ recovered the intact structure of nucleosomes (at least in the region of contact between two H2A-H2B dimers), which was partially disrupted by the removal of histone tails. The stabilizing effect of FACT_-N_ was detected for both nucleosomes with disturbed DNA folding (high-E_PR_ fraction) and unfolded nucleosomes (low-E_PR_ fraction).

In contrast, the addition of FACT_-N_(SSRPΔ) to tailless nucleosomes (Figure 4B) resulted in minimal, non-significant changes in the low-E_PR_ fraction (*p* = 0.36) and the position of the high-E_PR_ maximum (*p* = 0.29). Thus, FACT_-N_(SSRPΔ) does not retain the nucleosome-stabilizing activity of FACT_-N_; the CTD and/or HMG domains of SSRP1 are essential for maintaining this activity of FACT.

## 3. Discussion

**Figure 5:**
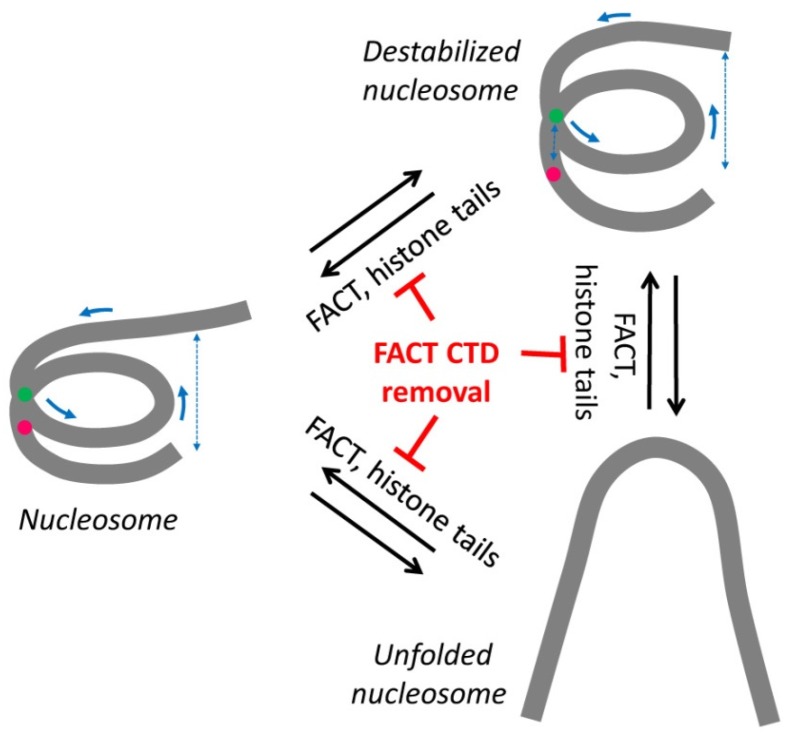
A model of FACT action during nucleosome stabilization. Histone tail removal results in destabilization of nucleosomes accompanied by partial uncoiling of the nucleosomal DNA. On a small fraction of the templates, nucleosomes are dramatically unfolded. The three transitions in the structure of the nucleosomes are reversible and likely require the presence of CTD domains on both FACT subunits; CTD removal blocks the transitions.

The tail domains of histones are important for the stability of the nucleosome core [25]. Results of our experiments (Figure 2C) showed that the removal of the tails resulted in moderate alterations in the DNA folding in the majority of nucleosomes and partially reversible unwrapping of DNA in a small fraction of nucleosomes (Figure 5), suggesting that histone tails stabilize the folding of nucleosomal DNA. It was shown that the removal of histone tails did not induce sliding of DNA relative to the histone octamer when strong nucleosome-positioning DNA sequences were used for nucleosome assembly [25]. Thus, the shift of the high-E_PR_ maximum in tailless nucleosomes (Figure 2C) was likely a result of the destabilization of folding of nucleosomal DNA and involved an increase in the distance between neighboring DNA gyres and possibly the weakening of some DNA-histone contacts (Figure 5). In summary, fluorescently labeled tailless nucleosomes can serve as an experimental model for analysis of both stabilizing and destabilizing activities of different protein factors using spFRET microscopy.

Our data also suggest that the CTD and/or HMG domains of SSRP1 subunit are required for the nucleosome-stabilizing activity of FACT. At the same time, unlike yFACT [18], human FACT_-N_ or FACT_-N_(SSRP∆) do not considerably affect the structure of intact nucleosomes (Figure 3B). Notably, in yFACT, SSRP1-like domains are distributed between two proteins: the DNA-binding high mobility group box (HMGB)-like domain is present in the non-histone protein 6 (Nhp6) subunit and the other homologous domains the in Pob3 subunit [26]. Using spFRET microscopy, we found previously that yFACT induces extensive, reversible unwrapping of nucleosomal DNA [18]. FACT_-N_ does not change the nucleosome structure at the concentration of 0.1 µM (Figure 3B). The effect of yFACT (nucleosomal DNA uncoiling) was observed at the range concentration of Spt16/Pob3 from 0.033 to 0.13 µM and Nhp6 from 0.33 to 1.3 µM. Thus, at similar concentrations of Spt16 and SSRP1/Pob3 subunits, FACT_-N_ and yFACT stabilize and considerably unfold nucleosomes, respectively.

The nucleosome reorganization by yFACT results in a strong increase of sensitivity of nucleosomal DNA to restriction enzymes, DNases and hydroxyl radicals [27,28]. This unwrapping requires the presence of DNA-binding subunit Nhp6, while the Spt16/Pob3 complex alone cannot destabilize nucleosomes [26]. The presence of several molecules of Nhp6 protein is required for nucleosome reorganization by yFACT [18,28]. Notably, nucleosomes containing DNA with a double-strand break are also reorganized by human FACT [17]. These studies suggest that human FACT more often works in a way similar to yFACT in the absence of the Nhp6 subunit, and has an intrinsic nucleosome-stabilizing but not nucleosome unfolding activity. A similar nucleosome-stabilizing activity of human FACT is essential for nucleosome survival during transcription elongation [5,8].

## 4. Materials and Methods

### 4.1. Purification of human FACT

BL21(DE3) *Escherichia coli* cells were transformed with PET-based expression plasmids encoding His-MBP-tagged Spt16 (501–1006) and SSRP1 (1–709) and cultured at 37 °C to an optical density of a sample measured at a wavelength of 600 nm (OD_600_) of ~0.1. Protein production was then induced with 0.5 mM isopropyl-β-D-1-tiogalactopyranoside (IPTG) and cells grown at 15 °C for 16–20 h using Innova 40R incubator shaker (Eppendorf, Hamburg, Germany). Cells were harvested by centrifugation at 4000 rpm for 20 min in a centrifuge 5810R (Eppendorf, Hamburg, Germany) and resuspended with 10 mL of bind buffer (50 mM NaH_2_PO_4_-Na_2_HPO_4_, 300 mM NaCl at pH 7.5). The cells were lysed using a high-pressure homogenizer Emulsiflex C5 (Avestin Inc., Ottawa, ON, Canada) and sonication. The lysate was centrifuged at 20,000 rpm for 45 min and the supernatant applied onto a Nickel-NTA column (Qiagen, Hilden, Germany) pre-equilibrated with bind buffer at 4 °C. The column was washed with 200 mL of wash buffer (50 mM NaH_2_PO_4_-Na_2_HPO_4_, 300 mM NaCl, 20 mM imidazole at pH 7.5) prior to protein elution with ~50 mL of elution buffer (50 mM NaH_2_PO_4_-Na_2_HPO_4_, 300 mM NaCl, 200 mM imidazole at pH 7.5). The His-MBP tag on Spt16 was cut using Tobacco Etch Virus (TEV) protease at 4 °C overnight in ThermoStat C (Eppendorf, Hamburg, Germany). Spt16 was then dialyzed against bind buffer to remove imidazole and separated from His-MBP using size exclusion chromatography with a HiLoad Superdex 200 column (GE Healthcare Bio-Sciences, Pittsburgh, PA, USA). Purified Spt16 and His-MBP-tagged SSRP1 were mixed at a 1:1 molar ratio and the complex purified using Nickel-nitrilotriacetic acid (Nickel-NTA) chromatography. SSRP1 His-MBP tag was cut with TEV protease at 4 °C overnight and the sample dialyzed against bind buffer to remove imidazole. The cleaved His-MBP tag was separated from FACT (i.e., Spt16-SSRP1) using Nickel-NTA chromatography. Fractions containing FACT were further purified by size exclusion chromatography using a HiLoad Superdex 200 column (GE Healthcare Bio-Sciences, Pittsburgh, PA, USA). The following versions of human FACT were purified: FACT_-N_, FACT_-N_ without CTD and HMG domains of SSRP1 (FACT_-N_(SSRP∆)) and FACT_-N_ without both acidic domains (CTD of Spt16 and CTD-HMG of SSRP1, FACT_-N_(Spt6∆/SSRP∆)) (see Results).

### 4.2. DNA Templates

DNA templates for nucleosome assembly were amplified by PCR using fluorescently-labeled primers forward 5′-ACCCCAGGGACTTGAAGTAATAAGGACGGAGGGCCT^#^CTTTCAACATCGAT (where T^#^—is a nucleotide labeled with ROX) and reverse 5′-CAAGCGACACCGGCACTGGGCCCGGTTCGCGCTCCCTCCTTCCGTGTGTTGTCGT*CTCT (where T*—is a nucleotide labeled with FAM).

A plasmid pDS containing the modified nucleosome high affinity sequence 603–42 [20] was used as a template. PCR products were purified with a QIAquick PCR Purification Kit (Qiagen, Hilden, Germany) following the manufacturer’s protocol.

### 4.3. Purification and Tryptic Cleavage of the Donor Chromatin

Donor chromatin without linker histones was purified from chicken erythrocytes as described [29]. The tryptic cleavage reaction mixture contained 3.2 mg/mL of chromatin (DNA concentration) and 0.2 mg/mL of trypsin. The reaction was performed in a buffer containing 10 mM Tris-HCl pH 7.5, 0.5 mM EDTA, 350 mM NaCl for 60 min at 25 °C. Aprotinin (0.34 mg/mL) was added to stop the digestion. The extent of digestion was analyzed using Laemmli electrophoresis in 18% SDS–polyacrylamide gel as described [30]. Proteins were visualized in the gel using 0.0025% solution of Coomassie R-250 in 50% EtOH and 10% acetic acid.

### 4.4. Nucleosome Assembly and Purification

Nucleosomes were assembled by histone octamer transfer from the donor chromatin by dialysis against decreasing concentrations of NaCl as described [29]. Nucleosomes were purified from the octamer exchange reaction mixture and analyzed in a native gel as described [18].

### 4.5. Incubation of the Nucleosomes with FACT

Formation of FACT complexes with nucleosomes was performed in a buffer containing 17 mM HEPES pH 7.6, 2 mM Tris-HCl pH 7.5, 0.8 mM Na_3_EDTA, 0.11 mM 2-merсaptoethanol, 11 mM NaCl, 1.1% glycerin, 12% sucrose as proposed previously [28]. FACT_-N_ and its mutant variants were used at final concentrations of 0.1 µM. Nucleosomes were added at a final concentration of 0.4 nM. Reaction mixtures were incubated for 10 min at 30 °C and analyzed by spFRET microscopy.

### 4.6. spFRET Measurements

spFRET measurements in solution and calculations were performed as described [22]. Briefly, the Confocor-3 module of the LSM710-Confocor3 laser scanning confocal microscope (Carl Zeiss AG, Oberkochen, Germany) was utilized to measure freely diffusing single nucleosomes. Measurements were performed using the C-Apochromat water-immersion 40× objective with the 1.2 numerical aperture (Carl Zeiss AG, Oberkochen, Germany). The fluorescence was excited with Ar^+^-laser using the 488 nm wavelength and detected within the 505–590 and >590 nm wavelength ranges for FAM and ROX, respectively. The measurements were typically conducted for 10–15 min.

The E_PR_ was calculated as

*E_PR_ = (I_Aa_ - α × I_Dd_)/(I_Aa_ + (1 - α) × I_Dd_),* (1)

where *I*_Aa_ is ROX fluorescence intensity in the ROX detection channel, *I*_Dd_ is FAM fluorescence intensity in the FAM detection channel (both corrected for background), α is the contribution of FAM fluorescence in the ROX detection channel (spectral cross-talk) calculated as

α = *I*_Da_*/I*_Dd_, (2)

where *I*_Da_ is FAM fluorescence intensity in the ROX detection channel corrected for background. For this pair of labels α = 0.1. Proximity ratios E_PR_ were calculated for (0.5–10) × 10^3^ single nucleosomes for each experimental sample and presented as a relative frequency distribution plot fitted with a sum of two Gaussians (goodness of fit *R*^2^ = 0.90–0.97). Fractions of nucleosomes differed by E_PR_ were calculated as the areas under the Gaussian peaks. Reproducibility of the results was verified in at least two independent experiments. Repeated measurements were used to calculate positions of E_PR_ maxima and percentage of nucleosomes in the fractions as mean ± 90% confidential interval.

## 5. Conclusions

In summary, our studies suggest that the removal of histone tails by trypsin disturbs the nucleosome structure and causes unwrapping of nucleosomal DNA which can be observed near the contact of the DNA between H2A-H2B dimers. Human FACT is able to stabilize the structure of tailless nucleosomes by reducing DNA unwrapping; the CTD and/or HMG domains of SSRP1 are required for this stabilization.

## Figures and Tables

**Table 1:** Positions of E_PR_ maxima and distribution of nucleosomes between two subpopulations as measured by spFRET microscopy for intact and tailless nucleosomes as well as for their complexes with FACT and FACT (SSRPΔ).

	N		N+FACT_-N_		FACT_-N_(SSRPΔ)		N(tailless)		N(tailless)+FACT_-N_		N(tailless)+FACT_-N_(SSRPΔ)	
Gaussians	Peak 1	Peak 2	Peak 1	Peak 2	Peak 1	Peak 2	Peak 1	Peak 2	Peak 1	Peak 2	Peak 1	Peak 2
E_PR_ (max)	0.07 ± 0.05	0.57 ± 0.01	0.06 ± 0.05	0.58 ± 0.01	0.08 ± 0.05	0.57 ± 0.01	0.08 ± 0.03	0.49 ± 0.01	0.06 ± 0.03	0.54 ± 0.01	0.08 ± 0.03	0.51 ± 0.01
Subpopulation (%)	9.3 ± 1.4	91 ± 2	7 ± 6	93 ± 6	9 ± 3	92 ± 3	20 ± 3	80 ± 3	8 ± 6	93 ± 9	17 ± 2	83 ± 2

## Supplementary Materials

The following are available online at http://www.mdpi.com/2072-6694/9/1/3/s1. Figure S1. Comparison of intact (A) and tailless (B) N35/112 nucleosomes.
