# Supplementary material for: Stabilization of Nucleosomes by Histone Tails and by FACT Revealed by spFRET Microscopy
Source: Cancers (Basel). 2017 Jan 6;9(1):3. doi: 10.3390/cancers9010003 (PMC5295774; doi:10.3390/cancers9010003)
Supplement: Supplementary file 1 [file cancers-09-00003-s001.pdf]

Supplementary Materials: Stabilization of Nucleosome by Histone Tails and by FACT Revealed by spFRET Microscopy

Maria E. Valieva, Nadezhda S. Gerasimova, Kseniya S. Kudryashova, Anastasia L. Kozlova, Mikhail P. Kirpichnikov, Qi Hu, Maria Victoria Botuyan, Georges Mer, Alexey V. Feofanov and Vasily M. Studitsky

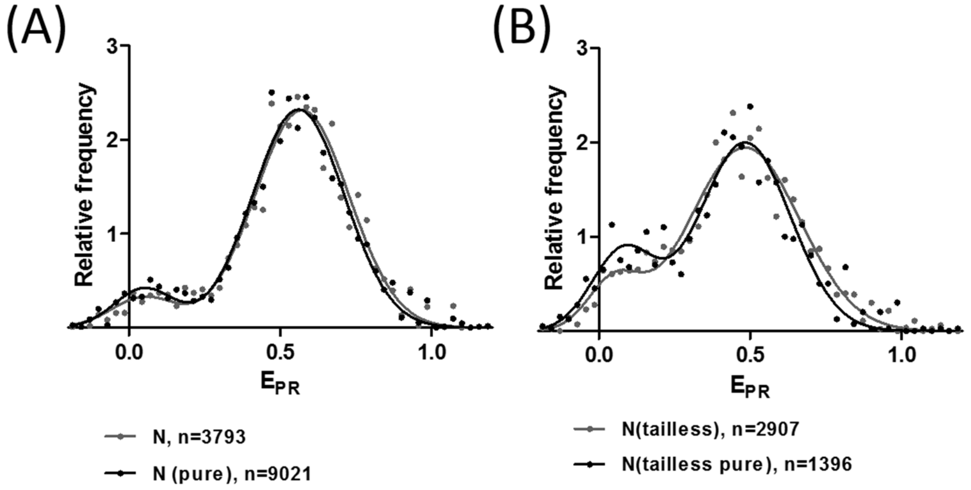

Figure S1. Comparison of intact (**A**) and tailless (**B**) N35/112 nucleosomes. Typical frequency distributions of proximity ratios (E_{PR}). Analysis by spFRET microscopy. The data collected in the presence of donor chromatin used for reconstitution and gel-purified (pure) nucleosomes (N) are shown. *n* is the number of analyzed nucleosomes.
